# MicroRNA-29a Is a Candidate Biomarker for Alzheimer’s Disease in Cell-Free Cerebrospinal Fluid

**DOI:** 10.1007/s12035-015-9156-8

**Published:** 2015-04-21

**Authors:** Mareike Müller, Lieke Jäkel, Ilona B. Bruinsma, Jurgen A. Claassen, H. Bea Kuiperij, Marcel M. Verbeek

**Affiliations:** 1 Department of Neurology, Donders Institute for Brain, Cognition and Behaviour, Radboud Alzheimer Center, Radboud University Medical Center, Nijmegen, The Netherlands; 2 Department of Laboratory Medicine, Radboud University Medical Center, P.O. Box 9101, 6500 HB Nijmegen, The Netherlands; 3 Department of Geriatric Medicine, Donders Institute for Brain, Cognition and Behaviour, Radboud Alzheimer Center, Radboud University Medical Center, Nijmegen, The Netherlands

**Keywords:** Alzheimer disease, microRNA, Biological marker, Cerebrospinal fluid, Diagnosis

## Abstract

The identification of reliable biomarkers for Alzheimer’s disease (AD) remains challenging. Recently, abnormal levels of microRNAs (miRNAs) miR-27a, miR-29a, miR-29b, and miR-125b in cerebrospinal fluid (CSF) of AD patients were reported. We aimed to confirm the biomarker potential of these miRNAs for AD diagnosis. Additionally, we examined the influence of blood contamination on CSF miRNA levels as potential confounding factor. We studied expression levels of the four miRNAs by quantitative PCR in CSF samples of AD patients and non-demented controls, and in blood-spiked CSF. Levels of miR-29a, but not of the other three miRNAs, were increased by a factor of 2.2 in CSF of AD patients. Spiking of small amounts of blood into CSF revealed that miR-27a and miR-29a, but not miR-125b levels were strongly influenced by the number of blood cells in the sample. In conclusion, miR-29a may be a candidate biomarker for AD, but only when used in cell-free CSF.

## Introduction

Alzheimer’s disease (AD) is the most common form of dementia worldwide. Diagnosis of AD is currently supported by measurement of total tau (t-tau), phosphorylated tau (p-tau), and amyloid-β42 (Aβ42) in cerebrospinal fluid (CSF). However, diagnostic accuracy of this panel of biomarkers has its limitations [1]. MicroRNAs (miRNAs) have been introduced as promising novel biomarkers. They are small, non-coding RNAs that can bind to specific mRNAs and regulate their expression by translational repression or degradation. Several miRNAs have been reported to regulate AD-associated proteins in the brain [2–4]. Furthermore, miRNAs appear to be very stable in body fluids [5–7] and even low concentrations are detectable by the widely used method of quantitative PCR (qPCR). Therefore, miRNAs are attractive targets in the search of novel biomarkers.

Altered expression of several miRNAs in CSF of AD patients has been previously reported. In two recent publications, it was reported that the levels of miR-27a and miR-125b are lower in CSF of AD patients than in controls [8, 9], whereas miR-29a and miR-29b levels are higher [9]. We aimed to confirm the potential of these miRNAs as biomarkers for AD. We previously showed that miRNA levels in CSF can be influenced by blood contamination [10]. Therefore, we also investigated whether traumatic lumbar puncture may influence CSF levels of these miRNAs. If so, this might influence outcomes of miRNA expression studies and the reliability of using these miRNAs as CSF biomarkers for AD.

## Methods

### Samples

CSF of 18 AD patients and 20 healthy individuals was obtained at the Radboud University Medical Center, Nijmegen. The samples had been collected by lumbar puncture in polypropylene tubes, centrifuged, aliquoted, and stored at -80 °C. AD patients had been diagnosed as probable AD according to the criteria of the National Institute of Neurological and Communicative Disorders and Stroke as well as Alzheimer’s Disease and Related Disorders Association (NINCDS/ADRDA), based on clinical evaluation, magnetic resonance imaging, and neuropsychological testing [11]. More recent diagnostic criteria [12] were not applied, since the patients’ samples were collected prior to the introduction of these criteria. However, most AD cases (14/18) had a typical CSF biomarker profile with abnormal levels of t-tau, p-tau, and Aβ42. In three cases, two of these biomarkers were abnormal, and in one case, one biomarker was abnormal. None of the AD patients had a CSF biomarker profile compatible with controls. Control subjects had been assessed for neurological disorders but were either diagnosed without a neurological disorder or with a systemic disease without neurological manifestations. CSF samples were obtained as part of the clinical diagnostic work-up of a patient. Patients were informed that their data, including CSF, could be used for further scientific purposes and were given the option to object against this use, in which case their data was not used. Only CSF samples with <4 leukocytes/μl and <200 erythrocytes/μl were selected. Controls and AD patients were from Western-European (Caucasian) origin, and were age- and sex-matched. An overview of patient and sample characteristics is given in Table 1.

**Table 1:** Group characteristics

	AD	Control	*p* value^a^
Number of patients	18	20	
Gender (male/female)	8/10	8/12	NS (*p* = 0.78)
Mean age (years)	70.4 ± 9.1	63.2 ± 12.3	NS (*p* = 0.05)
MMSE	19.7 ± 3.2	ND	NA
Number of erythrocytes/μl^b^	17.7 ± 49.0 (0 – 165)	3.0 ± 5.8 (0 – 21)	NS (*p* = 0.19)
Number of leukocytes/μl^b^	0.6 ± 0.9 (0 – 3)	0.4 ± 0.6 (0 – 2)	NS (*p* = 0.39)
Total protein (mg/L)^b^	566 ± 156 (374 – 976)	578 ± 212 (284 – 971)	NS (*p* = 0.85)

All data are expressed as mean ± standard deviation

Abbreviations: *AD* Alzheimer’s disease, *NS* not significant, *MMSE* Mini Mental State Examination, *ND* not determined, *NA* not applicable

^a^Gender distribution of AD and control groups were compared using the chi-square test, all other parameters using a two-tailed *t* test

^b^The minimum-maximum range of the number of erythrocytes, leukocytes, and total protein is indicated in parentheses

### Preparation of Blood-Spiked CSF

Fresh whole blood from a healthy individual was spiked into 500 μl aliquots of cell-free CSF to final concentrations of 10 to 20,000 erythrocytes/μl CSF. Cell count was confirmed using flow cytometry. Samples were incubated at room temperature for 3 h, followed by centrifugation to remove cells to imitate CSF processing in clinical circumstances. Likewise, the effect of incubation time was examined by applying incubation periods of 0 to 180 min between spiking of 20,000 cells/μl and centrifugation.

### RNA Isolation and Quantitative PCR

RNA isolation, reverse transcription, pre-amplification, and qPCR were performed as previously described by us [10]. Primer sequences of hsa-miR-27a-3p, hsa-miR-29a, hsa-miR-29b-2-5p, and hsa-miR-125b-5p can be found at http://appliedbiosystems.com.

### Data Analysis

To normalize expression levels of miRNAs, the geometric mean (GM) of three reference RNAs was used. Those had similar cycle threshold (Ct) values in the CSF of AD and control groups. The following formulas were used to calculate relative expression levels (RELs) and GM: REL = 2^-ΔCt^, with ΔCt = Ct_*miRNA*_ − GM, and $\mathrm{G}\mathrm{M}=\sqrt[3]{{\mathrm{CT}}_{\mathrm{miR}\hbox{-} 16}\cdot {\mathrm{CT}}_{\mathrm{miR}\hbox{-} 24}\cdot {\mathrm{CT}}_{\mathrm{U}6}}$. Data were analyzed using GraphPad Prism version 5 (La Jolla, CA, USA). Normally distributed RELs were compared using a two-tailed *t* test, and the Mann Whitney *U* test was used for non-parametric data. Correlations were determined using Pearson *r*. Statistical outliers were identified using the Dixons Q test and the Grubbs test for outliers. *P* values were considered significant when *p* < 0.05. Receiver operator characteristics (ROCs) analysis was used to determine the diagnostic value of miR-29a and miR-125b. Area under the curve (AUC) was calculated with 95 % confidence intervals. The optimal cut-off value was determined using the Youden index (sensitivity + specificity − 1.0) and likelihood ratio (sensitivity/(1 − specificity)).

## Results

### MiRNA Levels in CSF

**Fig. 1:**
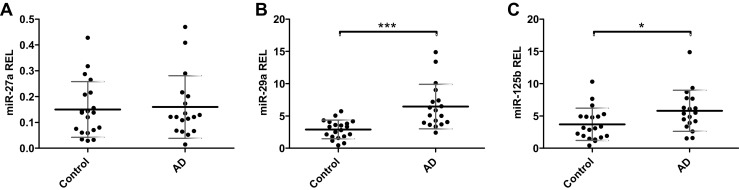
MiRNA levels in cell-free CSF of AD patients and controls. Relative expression levels (REL) of miR-27a (**a**), miR-29a (**b**), and miR-125b (**c**) in CSF of AD patients and control subjects. Data are presented as scatter plots with mean REL value ± standard deviation indicated. Key: *p < 0.05; ***p < 0.001

We measured miR-27a, miR-29a, miR-29b, and miR-125b levels in (nearly) cell-free CSF samples of AD patients and controls. Numbers of leukocytes and erythrocytes and total protein levels did not differ between groups (Table 1). MiR-29b could only be detected in five AD and five control samples and was therefore excluded from further analysis. The other three miRNAs were detectable in all samples. Mean miR-27a levels were similar in AD and control samples (Fig. 1a), but mean miR-29a levels were increased by a factor of 2.2 in the AD group compared to controls (*p* = 0.0001, Fig. 1b) and differentiated AD from controls with a sensitivity of 89 % and a specificity of 70 % (cut-off > 3.61; area under the curve (AUC) = 0.87, likelihood ratio = 2.96, Youden index = 0.59). Mean miR-125b levels were slightly increased in AD (*p* = 0.025, Fig. 1c) and differentiated between AD and controls with a sensitivity of 78 % and a specificity of 60 % (cut-off > 3.64; AUC = 0.71, likelihood ratio = 1.94, Youden index = 0.38). However, this difference mainly depended on one statistical outlier in the AD group. Without this outlier, the difference in expression was no longer significant (*p* = 0.058).

### Effect of Blood Contamination on CSF miRNA Levels

**Fig. 2:**
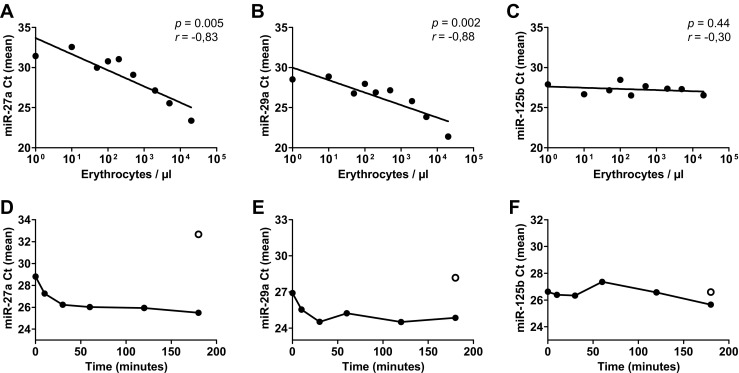
Effect of spiking cell-free CSF with whole blood. Cell-free CSF was spiked with different volumes of whole blood. After centrifugation, miR-27a (**a**), miR-29a (**b**), and miR-125b (**c**) levels were analyzed. Also, the effect of incubation period with blood cells on CSF miRNA levels was measured. Spiked blood cells were removed from CSF after various periods of time and expression levels of miR-27a (**d**), miR-29a (**e**), and miR-125b (**f**) were measured. Data are represented by cycle threshold (Ct) values. Key: *empty circle* = cell-free CSF; *filled circle* = blood-spiked CSF

We studied the effect of blood contamination, which often occurs during lumbar puncture of CSF, on miRNA levels. We simulated this effect by spiking whole blood into cell-free CSF. Mean Ct values of miR-27a and miR-29a were negatively correlated with the numbers of erythrocytes (miR-27a: *r* = –0.89, *p* = 0.001, Fig. 2a; miR-29a: *r* = –0.88, *p* = 0.002, Fig. 2b), indicating a strong dependence of these miRNA levels on the number of blood cells present in the CSF sample. No correlation was evident for miR-125b (Fig. 2c). We also investigated how fast the effect of blood contamination on miRNA levels occurred. A comparison of the Ct values for miR-27a and miR-29a in cell-free and blood-spiked CSF samples showed that mean Ct values for these miRNAs in CSF decreased, and thus miRNA levels increased, immediately after blood-spiking (Fig. 2d, e). A plateau was reached after 30 min. As expected, miR-125b levels were not affected by blood contamination at any time duration of incubation (Fig. 2f).

## Discussion

In AD, many miRNAs (e.g. miR-9, miR-146, miR-107, miR-124) have been described that are differentially expressed in the brain and regulate proteins involved in AD pathogenesis (see for an overview [13] and [14]). A subset of miRNAs can also be detected in CSF. Therefore, these miRNAs have been suggested as potential diagnostic tools for AD, provided that their levels can be quantified in volumes that are suitable for diagnostic purposes (<1 ml) [8–10]. In an earlier study, we investigated several miRNAs of interest in hippocampus and CSF, but only few miRNAs (miR-16 and miR-146a) were detectable in CSF, and furthermore, we discovered that the levels of miR-146a are influenced by blood contamination [10]. With this study, we aimed to investigate other recently proposed miRNA biomarkers in CSF, and tested whether miR-27a, miR-29a, miR-29b, and miR-125b (previously reported with different levels in CSF of AD patients compared to controls [9, 8]) may indeed serve as biomarkers for AD.

We found similar levels of miR-27a in CSF samples of AD patients and controls, which contradicts the previously reported decrease of miR-27a levels in AD CSF [8]. A possible explanation for this discrepancy is the effect of blood contamination on miR-27a CSF levels as we demonstrated in this study. However, since no information on cell count was provided in the earlier study, our assumption remains speculative. MiR-29a levels in CSF of AD patients were significantly increased compared to controls and discriminated AD from controls with good sensitivity and moderate specificity. The increased miR-29a levels in AD confirm previous findings [9] and are also in line with a report on its increased levels in the medial frontal gyrus of AD patients [15]. In contrast, another study reported decreased miR-29a levels in the cortex of AD patients [16]. Interestingly, BACE1, an enzyme involved in proteolytic production of the Aβ protein from its precursor protein, is regulated by miR-29a [2]. A potential drawback for the biomarker potential of CSF miR-29a levels may be that its levels are strongly correlated to the number of blood cells if present in the CSF, as we show in this study. Our finding of slightly increased miR-125b levels in CSF of AD patients compared to controls is in contrast to a previous study in which a decrease was reported [9]. The increase we found was mainly dependent on one AD sample, and should therefore be interpreted with caution. Our findings are, however, consistent with the reported levels of miR-125b in brain tissue, i.e. an increase in hippocampus, medial frontal gyrus, and temporal cortex of AD patients [15, 17]. If additional studies can confirm the increased levels of miR-125b in CSF of AD patients, its potential as a diagnostic marker would be strengthened by the fact that miR-125b levels are not influenced by blood contamination.

MiRNAs are found in plasma and in serum [9, 18], and in several cell types present in blood (e.g. blood mononuclear cells, erythrocytes, or leukocytes [19–21]). It is therefore possible that these are the sources of blood-derived miRNAs that are released in the CSF upon traumatic lumbar puncture, hence influencing miRNA levels. The effect of blood contamination on miR-27a and miR-29a levels in CSF occurred very fast. Their levels are altered within 10 min after blood spiking, which means that contamination cannot be prevented by fast processing of samples. Thus, these miRNAs cannot serve as biomarkers in CSF collected after a traumatic lumbar puncture.

This study demonstrated that reproducing previously reported CSF miRNA expression data can be challenging and that blood contamination may be a major factor that influences miRNA levels. Hereby, blood-derived miRNAs may alter the levels in CSF, but possibly also other factors such as miRNA degrading enzymes present in the blood may play a role in influencing miRNA CSF levels. For our study, we also cannot exclude that the mean difference in age, although not statistically significant, between the AD and the control group may have biased our results. This, however, requires further study. Other factors that may account for contradictory miRNA expression data are normalization methods [22] and cohort size. Also, degeneration of the brain in AD might be an important factor that could lead to decreased levels of some miRNAs, rather than specific downregulation. However, this does not account for the miRNAs that were investigated in this study, which were either upregulated or were present at equal levels in AD patients compared to controls. Further, there is increasing evidence that etiology plays an important part in genetic variation, including variations in miRNA-encoding DNA, and that expression profiles are population-specific and therefore could lead to different results across different populations [23, 24]. Despite these considerations, miR-29a may be a promising biomarker for AD. However, significantly larger studies in patients with different genetic backgrounds will be necessary to further validate miR-29a as an AD biomarker. This study investigated four miRNAs, but there may be more miRNAs that could serve as biological markers in CSF, either stand-alone or as part of a miRNA panel. In addition, miRNAs may be useful biomarkers for AD severity and should be tested in different stages, for instance in patients with mild cognitive impairment due to AD compared to AD patients with different CDR scores.

## Abbreviations

Aβ42: Amyloid-β42
AD: Alzheimer’s disease
AUC: Area under the curve
CSF: Cerebrospinal fluid
Ct: Cycle threshold
GM: Geometric mean
miRNAs: microRNAs
NINCDS/ADRDA: National Institute of Neurological and Communicative Disorders and Stroke/Alzheimer’s Disease and Related Disorders Association
p-tau: Phosphorylated tau
qPCR: Quantitative PCR
REL: Relative expression level
ROC: Receiver operator characteristics
t-tau: Total tau
